# Association of Insurance Status with Severity and Management in ED Patients with Asthma Exacerbation

**DOI:** 10.5811/westjem.2015.11.28715

**Published:** 2016-01-12

**Authors:** Kohei Hasegawa, Samantha J. Stoll, Jason Ahn, Rashid F. Kysia, Ashley F. Sullivan, Carlos A. Camargo

**Affiliations:** * Massachusetts General Hospital, Harvard Medical School, Department of Emergency Medicine, Boston, Massachusetts; † North Shore Medical Center, Department of Emergency Medicine, Salem, Massachusetts; ‡ John H. Stroger Jr. Hospital of Cook County, Department of Emergency Medicine, Chicago, Illinois

## Abstract

**Introduction:**

Previous studies have demonstrated an association of low socioeconomic status with frequent asthma exacerbations. However, there have been no recent multicenter efforts to examine the relationship of insurance status – a proxy for socioeconomic status – with asthma severity and management in adults. The objective is to investigate chronic and acute asthma management disparities by insurance status among adults requiring emergency department (ED) treatment in the United States.

**Methods:**

We conducted a multicenter chart review study (48 EDs in 23 U.S. states) on ED patients, aged 18–54 years, with acute asthma between 2011 and 2012. Each site underwent training (lecture, practice charts, certification) before reviewing randomly selected charts. We categorized patients into three groups based on their primary health insurance: private, public, and no insurance. Outcome measures were chronic asthma severity (as measured by ≥2 ED visits in one-year period) and management prior to the index ED visit, acute asthma management in the ED, and prescription at ED discharge.

**Results:**

The analytic cohort comprised 1,928 ED patients with acute asthma. Among these, 33% had private insurance, 40% had public insurance, and 27% had no insurance. Compared to patients with private insurance, those with public insurance or no insurance were more likely to have ≥2 ED visits during the preceding year (35%, 49%, and 45%, respectively; p<0.001). Despite the higher chronic severity, those with no insurance were less likely to have guideline-recommended chronic asthma care – i.e., lower use of inhaled corticosteroids (ICS [41%, 41%, and 29%; p<0.001]) and asthma specialist care (9%, 10%, and 4%; p<0.001). By contrast, there were no significant differences in acute asthma management in the ED – e.g., use of systemic corticosteroids (75%, 79%, and 78%; p=0.08) or initiation of ICS at ED discharge (12%, 12%, and 14%; p=0.57) – by insurance status.

**Conclusion:**

In this multicenter observational study of ED patients with acute asthma, we found significant discrepancies in chronic asthma severity and management by insurance status. By contrast, there were no differences in acute asthma management among the insurance groups.

## INTRODUCTION

Asthma prevalence remains at historically high levels, affecting 26 million Americans in 2011.^1^ Asthma exacerbations were responsible for approximately two million emergency department (ED) visits in 2012.^2^ ED visits for asthma exacerbation suggest a failure of prevention-oriented care since most asthma exacerbations are preventable with high-quality longitudinal management.^3^,^4^ The morbidity burden is uneven in patients presenting to the ED with asthma exacerbation – the population at high risk. In a previous multicenter study of ED patients with asthma exacerbation during 1997–1998,^5^ we found that uninsured adults received suboptimal chronic asthma care (e.g., lower inhaled corticosteroid [ICS] use) and had higher chronic asthma severity (e.g., frequent ED visits). Despite a substantial ongoing burden of asthma-related ED visits, there have been no recent efforts to examine chronic and acute asthma management disparities by insurance status in this population. To address the knowledge gap, using data from a multicenter observational study of ED patients with asthma exacerbation, we investigated whether chronic asthma severity, guideline-recommended chronic asthma care, and acute asthma management differ by insurance status.

## METHODS

This is a secondary analysis of data from a multicenter chart review study that characterized adult ED patients with asthma exacerbation, as part of the Multicenter Airway Research Collaboration (MARC).^6^ The study setting, methods of measurement, and collected variables have been reported previously.^7^–^13^ Briefly, we recruited EDs by inviting sites that had participated in the earlier MARC study that evaluated ED patients with asthma exacerbation during 1996 to 2001.^5^,^14^,^15^ A total of 48 EDs across 23 U.S. states completed the current study.

Using the ICD-9-CM code 493.xx,^16^ each site identified all visits with a primary ED or hospital discharge diagnosis of asthma during a 12-month period, from January 1, 2011, to December 31, 2012 (i.e., sites had 24-month window from which to select the 12-month study period) among patients aged 18 to 54 years with a history of physician-diagnosed asthma. Onsite chart abstractors reviewed medical records (ED, inpatient, primary care physician, and/or specialist records) of 40 patients who were randomly selected by the EMNet Coordinating Center at Massachusetts General Hospital. In the case of repeat visits, we only included the first randomly sampled ED visit. This approach was also used in other MARC studies.^17^,^18^ Two hospitals each examined an additional 40 randomly selected patients to obtain a total of 2,000 patients.

Data abstraction was performed with a standardized form and included patients’ demographics, primary insurance type, estimated household income, chronic asthma factors, current asthma medications, specialty care status, details of the current asthma exacerbation, asthma management in ED or at discharge, and disposition. We estimated household income using home ZIP codes.^19^ Specialty care was defined as outpatient asthma care by an allergist/immunologist, pulmonologist, or other physician specifically focusing on asthma care (e.g., a general internist who is director of the local asthma center). All abstracters were trained with a one-hour online lecture, followed by the completion of two practice medical records, which were evaluated with a “criterion standard.” If a reviewer’s accuracy was <80% per medical record, the reviewer was retrained.

The outcome measures were 1) chronic asthma severity (as measured by ≥2 ED visits in a one-year period);^7^,^20^ 2) guideline-recommended chronic asthma care (i.e., use of ICS and evaluation by an asthma specialist) prior to the index ED visit;^3^,^4^ and 3) acute asthma management (i.e., use of systemic corticosteroid in the ED and initiation of ICS at ED discharge). Frequency of ED visits with acute asthma during the preceding one year from the index ED visit was measured by reviewing the medical records (not only ED records but also inpatient, primary care physician, and/or specialist records) for each patient. Specialty care was defined as outpatient asthma care by an allergy/immunologist, pulmonologist, or other physician focusing on asthma care (e.g., a general internist who is director of the local asthma center).

For the purpose of this analysis, we classified patients into three groups based on their primary insurance status: private insurance, public insurance (e.g., Medicaid, Medicare), and no insurance.^5^,^14^,^15^ To examine the association of insurance status with each outcome, we constructed logistic regression models adjusting for age, sex, race/ethnicity, estimated household income, and history of hospitalization and intubation for asthma exacerbation. We used the generalized estimating equations to account for patient clustering within EDs. All analyses were performed with SAS 9.4 (SAS Institute, Cary, NC). The institutional review board of each participating hospital approved the study.

## RESULTS

Of 2,000 ED patients with asthma exacerbation, 1,928 patients (96%) had data on insurance status and were included in the analysis. The analytic and non-analytic cohorts were similar in their age, sex, chronic asthma factors and management, ED asthma management, and disposition (Appendix 1). Among the analytic cohort, 632 patients (33%) had private insurance, 775 (40%) had public insurance, and 521 (27%) had no insurance. Patient demographics differed across the groups (Table 1). For example, compared to patients with private insurance, those with no insurance were more likely to be male, non-Hispanic black and smoker, and less likely to have a primary care physician (all p<0.001).

Likewise, chronic asthma factors and management differed across the groups. Compared to patients with private insurance, those with public insurance or no insurance were more likely to have a marker of chronic asthma severity (i.e., ≥2 ED visits in a one-year period) (35%, 49%, and 45%, respectively; p<0.001; Table 1). Despite their higher chronic severity, those with no insurance were less likely to have received guideline-recommended chronic asthma care before the ED visit – i.e., lower use of ICS (41%, 41%, and 29%; p<0.001) and lower utilization of asthma specialist care (9%, 10%, and 4%; p<0.001). However, the proportion of patients who had received these two chronic asthma management measures was low across the groups.

By contrast, there were no clinically important differences in ED presentation or statistically significant differences in acute asthma management by insurance status (Table 1). However, even with the higher chronic asthma severity of patients with public insurance or no insurance, the proportion of patients newly prescribed ICS at ED discharge did not differ across the groups (12%, 12%, and 14%; p=0.57). In multivariable models adjusting for potential confounding factors and patient clustering, these results did not change materially (Table 2).

## DISCUSSION

In this multicenter study of ED adult patients with asthma exacerbation, we found significant discrepancies in chronic asthma severity and management by insurance status. Specifically, compared to patients with private insurance, those with public insurance or no insurance had a higher risk of frequent ED visits (≥2 visits during the preceding year). Yet, even with the higher chronic asthma severity, those with no insurance were less likely to have received guideline-recommended chronic asthma care. By contrast, ED asthma treatment did not differ across the insurance groups. However, at ED discharge, patients with public insurance or no insurance were, despite their much higher chronic severity, equally likely to be newly prescribed ICS.

Our findings were disappointingly similar to those of our previous multicenter study of 1,019 adult ED patients with asthma exacerbation in the late 1990s.^5^ With the use of a similar design and setting, the previous study found that uninsured patients received suboptimal longitudinal care prior to their ED visits, despite their higher chronic severity. These observations paralleled another multicenter study of 965 children presenting to the ED with asthma exacerbation in the same period.^15^ Additionally, in the community setting, studies have reported that patients with public insurance had fewer prescriptions of ICS,^21^ difficulties with scheduling outpatient care,^22^ and higher rates of ED visits for asthma exacerbation.^23^,^24^ To our knowledge, our multicenter study is the largest study to have investigated chronic and acute asthma management disparities by insurance status among ED patients. Our data corroborate these findings and extend them by demonstrating persistent disparity not only in the use of ICS but also in utilization of specialist care.

The reasons for the disproportionate healthcare-related disparity is likely multifactorial. Potential explanations include differences in patient demographics (e.g., race/ethnicity) and socioeconomic status (e.g., income). However, our inferences did not change even after adjusting for these factors. Alternatively, insurance status may be an identifiable surrogate marker for many patient and health system factors – e.g., patient’s health beliefs, self-management knowledge, and access to preventive care.^25^

## LIMITATIONS

This study has several potential limitations. First, we relied on chart review for data collection; therefore, error in data measurement is possible. For example, our methods of assigning insurance status may have led to misclassification. However, the use of medical records is likely to be more accurate than patient self-report.^26^ Additionally, although we did not measure interrater agreement in this study, we used a previously-applied standardized data collection system with uniform definitions and rigorous training, which achieved a high inter-observer agreement (*k* coefficients, 0.6–1.0) in our recent study.^27^ Second, as with any observational studies, the observed associations do not necessarily prove causality and might be confounded by unmeasured factors (e.g., access to ambulatory care, inter-hospital practice variations). However, we addressed this concern, at least partially, by accounting for clustering. Lastly, the EDs that composed this study were mainly urban, academic centers. This study setting resulted in a high proportion of patients with public insurance or no insurance as well as urban-dwelling minorities. However, our observations might not be generalized to rural, community EDs where asthma morbidity is also high.^28^

## CONCLUSION

In this large multicenter study of 1,928 ED patients with asthma exacerbation, we found an ongoing disparity in disease burden and management by insurance status. Compared to patients with private insurance, those with public insurance or no insurance had a greater risk of frequent ED visits. However, those with no insurance had less utilization of asthma controller medications and asthma specialist care. Although acute asthma treatment in the ED did not differ by insurance, patients with public insurance or no insurance were no more likely to be newly prescribed ICS at ED discharge despite their higher chronic severity. Additionally, we found that only a small subset of patients received guideline-recommended prevention-oriented asthma care regardless of their insurance. Therefore, it is unlikely that expanding insurance coverage through the Affordable Care Act alone can fully address the observed disparities in this high-risk population. Our data should encourage policymakers and clinicians to improve access to asthma specialists and promote greater implementation of the evidenced-based asthma guidelines.^3^,^4^

## Supplementary Information



## Figures and Tables

**Table 1:** Patient characteristics and emergency department course, according to primary insurance status.

Patient characteristics	Private insurance (n=632; 33%)	Public insurance (n=775; 40%)	No insurance (n=521; 27%)	P value
Demographics				
Age (y), median (IQR)	34 (25–45)	35 (25–45)	33 (25–45)	0.38
18–29	249 (39)	267 (34)	203 (39)	
30–39	145 (23)	207 (27)	129 (25)	
40–54	238 (38)	301 (39)	189 (36)	
Male sex	230 (36)	267 (35)	285 (55)	<0.001
Body mass index, median (IQR)*	31 (26–37)	31 (26–38)	28 (24–34)	<0.001
Race/ethnicity†				<0.001
Non-Hispanic white	170 (27)	107 (14)	99 (19)	
Non-Hispanic black	297 (47)	414 (53)	291 (56)	
Hispanics	103 (16)	184 (24)	88 (17)	
Other	30 (5)	20 (3)	8 (2)	
Median household income estimated from ZIP code, median (IQR)	$39,327 ($28,337–$57,004)	$32,733 ($25,967–$45,137)	$34,167 ($25,991–$46,377)	<0.001
Having primary care physician	450 (71)	506 (65)	189 (36)	<0.001
Active smoker	160 (25)	265 (34)	203 (39)	<0.001
Chronic asthma factors				
Ever hospitalized for asthma	202 (32)	314 (41)	151 (29)	<0.001
Ever intubated for asthma	70 (11)	123 (16)	47 (9)	0.01
ED visit for asthma in past 12 months	219 (35)	381 (49)	235 (45)	<0.001
Hospitalization for asthma in past 12 months	74 (12)	150 (19)	63 (12)	<0.001
Chronic asthma care				
Current use of oral corticosteroids	86 (14)	100 (13)	53 (10)	0.17
Current use of ICS	259 (41)	315 (41)	153 (29)	<0.001
Current use of long-acting β-agonist	168 (27)	213 (27)	87 (17)	<0.001
Current use of leukotriene modifiers	82 (13)	102 (13)	31 (6)	<0.001
Seen by asthma specialist in past 12 months	55 (9)	78 (10)	21 (4)	<0.001
ED presentations				
Duration of symptoms				
≤3 hours prior to ED arrival	53 (8)	78 (10)	54 (10)	0.40
Vital signs				
Initial respiratory rate (breaths/min), median (IQR)	20 (18–22)	20 (18–22)	20 (18–22)	0.04
Initial oxygen saturation (%), median (IQR)	98 (96–99)	98 (96–99)	97 (95–99)	0.01
Initial PEF (L/min), median (IQR)‡	240 (160–320)	230 (170–300)	235 (175–300)	0.81
Concomitant medical disorders§	94 (15)	119 (15)	51 (10)	0.01
ED treatment				
Inhaled β-agonists	626 (99)	769 (99)	516 (99)	0.92
Inhaled anticholinergics	434 (69)	571 (74)	380 (73)	0.09
Systemic corticosteroids	471 (75)	613 (79)	406 (78)	0.08
Intravenous magnesium	53 (8)	81 (10)	42 (8)	0.25
Mechanical ventilation	12 (2)	8 (1)	7 (1)	0.38
ED disposition				<0.001
Sent home	525 (83)	488 (76)	457 (88)	
Hospitalized	97 (15)	172 (22)	55 (11)	
Other (e.g., left against medical advice)	10 (2)	15 (2)	9 (2)	
ED length of stay (min), median (IQR)	183 (123–283)	188 (120–301)	175 (117–287)	0.52
Prescribed medications at ED discharge				
Prescribed oral corticosteroids¶	365 (70)	448 (76)	341 (75)	0.01
Newly prescribed ICS||	38 (12)	46 (12)	47 (14)	0.57

*IQR,* interquartile ratio; *ICS,* inhaled corticosteroids; *ED,* emergency department, *PEF,* peak expiratory flow

* Analyzed for 1,179 patients with body mass index available.

† Percentages are not equal to 100 because of missing data.

‡ Analyzed for 805 patients with initial PEF available.

§ Defined by pneumonia, congestive heart failure, pneumothorax, arrhythmia, sinusitis, and otitis media.

*ED,* emergency department; *IQR,* interquartile ratio; *ICS,* inhaled corticosteroids

¶ Analyzed for discharged patients (n=1,570).

|| Analyzed for discharged patients who did not report recent use of inhaled corticosteroids (n=1,042).

**Table 2:** Unadjusted and multivariable-adjusted associations of insurance status with study outcomes.

	Private insurance		Public insurance		No insurance	
	
Outcomes and models	OR (95% CI)	P value	OR (95% CI)	P value	OR (95% CI)	P value
ED visit for asthma in past 12 months						
Unadjusted model*	reference	-	1.79 (1.40–2.27)	<0.001	1.48 (1.13–1.94)	0.004
Adjusted model†	reference	-	1.64 (1.29–2.10)	<0.001	1.55 (1.17–2.05)	0.002
Current use of ICS						
Unadjusted model*	reference	-	0.97 (0.75–1.24)	0.79	0.58 (0.42–0.81)	0.001
Adjusted model†	reference	-	0.85 (0.67–1.08)	0.19	0.63 (0.45–0.86)	0.004
Evaluation by asthma specialist in past 12 months						
Unadjusted model*	reference	-	0.97 (0.68–1.38)	0.86	0.43 (0.28–0.65)	<0.001
Adjusted model†	reference	-	0.93 (0.64–1.36)	0.70	0.52 (0.32–0.84)	0.008
Use of systemic corticosteroids in the ED						
Unadjusted model*	reference	-	1.38 (1.10–1.74)	0.006	1.19 (0.94–1.51)	0.14
Adjusted model†	reference	-	1.25 (0.98–1.60)	0.07	1.27 (0.98–1.64)	0.07
Newly prescribed ICS at ED discharge‡						
Unadjusted model*	reference	-	0.74 (0.50–1.10)	0.14	0.86 (0.54–1.38)	0.54
Adjusted model†	reference	-	0.73 (0.49–1.07)	0.10	0.91 (0.57–1.46)	0.70

*OR,* odds ratio; *CI,* confidence interval; *ED,* emergency department; *ICS,* inhaled corticosteroids

* Unadjusted logistic regression model using the generalized estimating equations to account for patient clustering within EDs.

† Multivariable logistic regression model using the generalized estimating equations to account for patient clustering within EDs, with adjusting for age, sex, race/ethnicity, estimated household income, and history of hospitalization and intubation for asthma exacerbation.

‡ Analyzed for discharged patients who did not report recent use of inhaled corticosteroids (n=1,042).
