# Supplementary material for: Association of Insurance Status with Severity and Management in ED Patients with Asthma Exacerbation
Source: West J Emerg Med. 2016 Jan 12;17(1):22–7. doi: 10.5811/westjem.2015.11.28715 (PMC4729414; doi:10.5811/westjem.2015.11.28715)
Supplement: Supplementary file 1 [file wjem-17-22-s001.pdf]

## Appendix 1. Selected Patient Characteristics in the Analytic and non-Analytic Cohort

| Characteristics | Analytic cohort (n=1,928; 96%) | Non-analytic cohort (n=72; 4%) | P value |
|---|---|---|---|
| Age (y), median (IQR) | 34 (25-45) | 38 (27-48) | 0.22 |
| Male sex | 782 (41) | 32 (44) | 0.51 |
| Body mass index, median (IQR) | 30 (25-37) | 29 (24-40) | 0.57 |
| Race/ethnicity | | | |
| Non-Hispanic white | 376 (21) | 14 (21) | 0.06 |
| Non-Hispanic black | 1,002 (55) | 14 (37) | |
| Hispanic ethnicity | 375 (21) | 10 (26) | |
| Others | 58 (3) | 6 (16) | |
| Primary health insurance | | | - |
| Private | 632 (33) | - | |
| Public | 775 (40) | - | |
| No insurance | 521 (27) | - | |
| Active smoker | 628 (36) | 21 (35) | 0.33 |
| Ever admitted for asthma | 667 (54) | 13 (38) | 0.06 |
| Hospitalization for asthma in past 12 months | 287 (23) | 7 (21) | 0.71 |
| ED visit for asthma in past 12 months | 835 (46) | 29 (42) | 0.54 |
| Current use of oral corticosteroids | 239 (13) | 10 (14) | 0.72 |
| Current use of ICS | 727 (38) | 28 (39) | 0.84 |
| Current use of leukotriene modifiers | 215 (11) | 6 (8) | 0.45 |
| Duration of symptoms | | | |
| ≤ 3 hours prior to ED arrival | 185 (10) | 8 (13) | 0.55 |
| Vital signs | | | |
| Initial respiratory rate (breaths/min), median (IQR) | 20 (18-22) | 20 (18-22) | 0.11 |
| Initial oxygen saturation (%), median (IQR) | 98 (96-99) | 97 (95-98) | 0.08 |
| Initial PEF (L/min), median (IQR) | 235 (170-300) | 250 (280-300) | 0.99 |
| ED treatment | | | |
| Inhaled β-agonists | 1,911 (99) | 72 (100) | 0.64 |
| Systemic corticosteroids | 1,490 (78) | 43 (68) | 0.07 |
| ED disposition | | | |
| Admission to hospital | 324 (17) | 7 (10) | 0.10 |
| ED length of stay (min), median (IQR) | 184 (120-291) | 210 (135-317) | 0.26 |

ED, emergency department; ICS, inhaled corticosteroids; IQR, interquartile range; PEF, peak expiratory flow.
Data were expressed as n (%) unless otherwise indicated.
